# Systemic single‐cell analysis of GPP reveals treatment‐responsive and persistent immune signatures

**DOI:** 10.1002/ctm2.70753

**Published:** 2026-08-03

**Authors:** Soyoung Jeong, Seungbok Lee, Christine Suh‐Yun Joh, James G. Krueger, Hyun Je Kim, Seong Jin Jo, Jong‐Hee Chae

**Affiliations:** ^1^ Department of Biomedical Sciences Seoul National University Graduate School Seoul South Korea; ^2^ Genomic Medicine Institute Seoul National University Medical Research Center Seoul South Korea; ^3^ Department of Pediatrics Seoul National University College of Medicine Seoul National University Children's Hospital Seoul South Korea; ^4^ Department of Genomic Medicine Seoul National University Hospital Seoul South Korea; ^5^ Laboratory of Investigative Dermatology The Rockefeller University New York New York USA; ^6^ Department of Microbiology and Immunology Seoul National University College of Medicine Seoul South Korea; ^7^ Department of Dermatology Seoul National University Hospital Seoul South Korea; ^8^ Cancer Research Institute Seoul National University College of Medicine Seoul South Korea; ^9^ Interdisciplinary Program in Artificial Intelligence (IPAI) Seoul National University Seoul South Korea; ^10^ Department of Dermatology Seoul National University College of Medicine Seoul South Korea; ^11^ Institute of Human‐Environmental Interface Biology Medical Research Center Seoul National University Seoul South Korea

**Keywords:** generalised pustular psoriasis, IL‐12/23 blockade, IL‐17A blockade, single‐cell RNA sequencing, treatment response

Dear Editor,

Generalised pustular psoriasis (GPP) is a rare inflammatory skin disease characterised by dysregulation of the interleukin (IL)‐36 axis and systemic inflammation.^1^, ^2^ We recently reported single‐cell transcriptomic profiles of two sibling GPP patients treated with IL‐12/23 blockade (ustekinumab) or IL‐17A blockade (secukinumab), demonstrating molecular changes in the skin associated with clinical improvement.^3^ These changes were aligned with transcriptional shifts observed in the skin following IL‐36R blockade (spesolimab) treatment,^4^ supporting the presence of a pathogenic loop involving IL‐36, IL17, and IL‐12/23 in GPP skin lesions.^3^

Here, we investigated the systemic immune landscape of these patients carrying *IL36RN* mutations (c.28C>T; p.Arg10Ter and c.115+6T>C; p.Arg10ArgfsTer1) before and after treatment (Table S1), along with two unaffected, mutation‐negative siblings as controls (Figure 1A and B). Peripheral blood mononuclear cells (PBMCs) collected before and after biologic therapy were profiled using single‐cell RNA sequencing (scRNAseq) analysis (see Supporting Information for detailed methods). Using systemic scRNAseq analysis, we aimed to profile cell type‐specific molecular signatures across controls, GPP and post‐treatment samples, to distinguish treatment‐responsive inflammatory programs from persistent immune alterations that remain despite clinical improvement.

**FIGURE 1:**
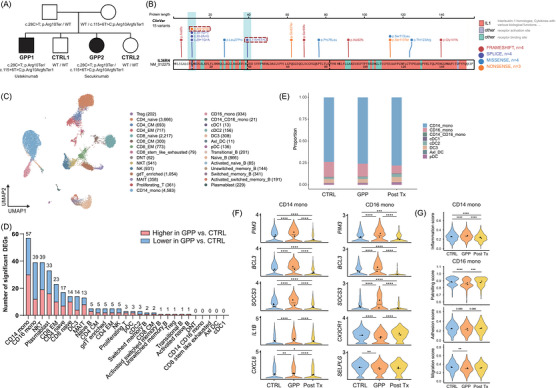
Characterisation of GPP patients compared to control and post‐treatment. (A) Pedigree chart showing familial relationships among study subjects. Squares represent males, and circles represent females. Filled symbols indicate GPP patients, and open symbols indicate unaffected individuals. (B) Lollipop diagram illustrating the distribution of GPP‐associated pathogenic or likely pathogenic variants in *IL36RN* reported in ClinVar. Frameshift, splice, missense and nonsense mutations are represented in pink, purple, blue and orange, respectively. (C) UMAP visualisation of 20,443 cells (Control, 6,607 cells; GPP, 6,697 cells; Post Tx, 7,139 cells). Clusters were annotated based on the expression of lineage markers. (D) Stacked bar plot showing the number of DEGs (avg log2FC > 0.5 or < –0.5 and adj *p* < 0.05) comparing control and GPP samples across cell types. (E) Stacked bar plot showing the relative proportions of DC and monocyte subsets within the total DC/monocyte compartment. (F) Stacked violin plots showing the expression of representative inflammatory genes in CD14 mono (left) and genes associated with immune surveillance and trafficking programs in CD16 mono (right). Black dots indicate sample level mean values. (G) Violin plots summarising inflammatory gene scores in CD14 mono and patrolling‐, adhesion‐, and migration‐associated gene scores in CD16 mono. Scores were calculated using UCell with the following gene lists: Inflammatory score (*IL1B*, *CXCL8*, *S100A8*, *SERPINB1*, *TNF*, *MARCO*, *CCL2*, *CD86*, *CD40*, *IL1RN*), patrolling score (*CX3CR1*, *FCGR3A*, *LST1*, *IFITM3*, *AIF1*, *SAT1*, *TYMP*, *FCER1G*), adhesion score (*ITGAL*, *ITGAM*, *ICAM3*, *PECAM1*, *SELL*, *FERMT3*), migration score (*CX3CR1*, *CCR5*, *CXCR4*, *RAC1*, *DOCK2*, *WAS*). Statistical comparisons were performed using the Wilcoxon rank sum test. *p* values were adjusted for multiple comparison using the Benjamini–Hochberg method. Individual cells were used as the unit of analysis, and black dots indicate sample‐level mean values. adj *p*, adjusted *p* value; avg log2 FC, average log2 fold change; CTRL, control; DC, dendritic cell; DEG, differentially expressed gene; GPP, generalised pustular psoriasis; mono, monocyte; Post Tx, post‐treatment; UMAP, uniform manifold approximation and projection.

A total of 20,443 cells were profiled and annotated (Figure 1C). Differential expression analysis revealed the highest numbers of differentially expressed genes (DEGs) in CD14 monocytes, which are primarily involved in inflammatory responses, and CD16 monocytes, which are specialised for vascular patrolling and immune surveillance (Figure 1D). Notably, monocytes and dendritic cells are known to express IL‐36R and IL1RAcP,^5^, ^6^ implicating these cells as major responders to IL‐36‐mediated inflammation in GPP.

To further delineate these alterations, we examined transcriptional changes within monocyte subsets, as cell proportions remained largely unchanged (Figures 1E and S1). In both CD14 and CD16 monocyte subsets, *PIM3*, *BCL3* and *SOCS3* were upregulated in GPP and decreased following treatment (Figure 1F), consistent with activation of inflammatory and cytokine‐responsive pathways. In CD14 monocytes, the expression of proinflammatory mediators *IL1B* and *CXCL8* was increased in GPP and reduced after treatment (Figure 1F). Accordingly, gene signature scoring revealed that inflammatory signatures were elevated in CD14 monocytes in GPP and decreased toward control levels after treatment (Figure 1G). In CD16 monocytes, *CX3CR1* and *SELPLG* were decreased in GPP and increased after treatment (Figure 1F). To further characterise transcriptional changes within this subset, we evaluated patrolling‐, adhesion‐, and migration‐associated gene signatures. These signatures were reduced in GPP and showed partial restoration following treatment (Figure 1G). These results suggest that GPP is associated with increased inflammatory activation in CD14 monocytes and altered transcriptional programs related to immune surveillance and trafficking in CD16 monocytes, both of which were partially restored following treatment with IL‐12/23 or IL‐17A blockade.

To gain deeper insight into the immune dysregulation in GPP, we examined the T cell compartment. Although overall proportions of major T cell subsets were broadly similar across groups, transcriptional analysis revealed differences in inflammatory and regulatory programs (Figures 2A and S1). In CD4 T cells, Th17‐associated signatures, including *RORC*, *CCR6*, *CCL20*, *STAT3* and *SOCS3*, were increased in GPP compared to controls (Figure 2B). In addition, *IL23R‐*expressing cells were observed primarily within CD4 naïve and central memory populations in GPP. While several of these expression changes declined after treatment, *RORC* expression in CD4 central memory and effector memory subsets, as well as *BATF* in effector memory cells, remained elevated after treatment (Figure 2B). Consistently, Th17 gene set scoring demonstrated elevated Th17 programs in CD4 central memory and effector memory T cells in GPP, with only partial normalisation after treatment (Figure 2C). Together, these findings indicate elevated Th17‐associated transcriptional programs in GPP that are not fully normalised following treatment despite clinical improvement.

**FIGURE 2:**
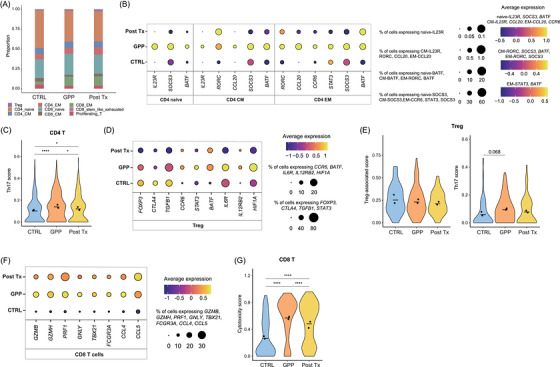
Characterisation of T cell subsets in GPP. (A) Stacked bar plot showing the relative proportions of T cell subsets as a total of T cells. (B) Dot plot showing expression of Th17‐associated genes across CD4 naive, central memory and effector memory T cell subsets. (C) Violin plot summarising Th17 signature score (*RORA*, *RORC*, *STAT3*, *CCR4*, *CCR6*) in CD4 central memory and effector memory T cells. (D) Dot plot showing expression of representative Treg‐associated genes and genes associated with Treg plasticity. (E) Violin plot summarising Treg‐associated signature score (*FOXP3*, *IL2RA*, *CTLA4*, *IKZF2*, *TIGIT*, *TNFRSF18*, *TGFB1*) and Th17 score (*RORA*, *RORC*, *STAT3*, *CCR4*, *CCR6*) in Tregs. (F) Dot plot illustrating expression of representative cytotoxicity genes in CD8 T cells. (G) Violin plot summarising cytotoxicity score (*PRF1*, *GZMB*, *GNLY*, *GZMH*, *TBX21*, *FCGR3A*, *CCL4*, *CCL5*) in CD8 T cells. Dot size represents the proportion of cells expressing each gene, and colour intensity indicates average expression level. Scores were calculated using UCell, and black dots indicate sample‐level mean values. Statistical analysis was conducted using the Wilcoxon rank sum test. *p* values were adjusted for multiple comparisons using the Benjamini–Hochberg method. CM, central memory; CTRL, control; EM, effector memory; GPP, generalised pustular psoriasis; Post Tx, post‐treatment; Treg, regulatory T cell.

As regulatory T cell (Treg) dysfunction has been implicated in psoriasis,^7^, ^8^ we next analysed the phenotype of circulating Tregs. In GPP, canonical Treg genes, *FOXP3*, *CTLA4* and *TGFB1*, were decreased, and remained low despite clinical improvement following treatment (Figure 2D). In contrast, genes associated with inflammatory activation and Treg plasticity, including *CCR6*, *STAT3*, *IL12RB2* and *HIF1A*, were upregulated in GPP (Figure 2D). Consistent with the transcriptional changes, Treg suppressive gene signature score tended to decrease in GPP and remained low after treatment, while Th17 gene signature score was increased in Tregs from patients with GPP and showed a modest decrease following treatment (Figure 2E). These findings suggest altered Treg‐associated transcriptional programs in GPP that are not fully normalised following treatment.

Given the observed alterations in Treg‐associated programs, we next examined the CD8 T cell compartment. Notably, genes involved in CD8 T cell cytotoxicity, including *GZMB*, *PRF1*, *GNLY*, *TBX21* and *CCL5*, were increased in CD8 T cells in GPP compared to controls (Figure 2F). Consistent with these findings, the CD8 cytotoxicity gene signature score was elevated in GPP and remained elevated following treatment (Figure 2G). Together, these results suggest persistence of cytotoxic CD8 T cell‐associated transcriptional programs that did not fully normalise despite clinical improvement.

In summary, systemic immune profiling revealed a dichotomy between treatment‐responsive innate inflammatory signatures in monocytes and persistent immune alterations within T cell subsets, including residual Th17‐associated signatures in CD4 T cells, altered Treg‐associated programs, and sustained cytotoxic CD8 T cell signatures. This longitudinal analysis provides insights into systemic immune dysregulation in paediatric familial GPP and its modulation following clinical improvement. These results are consistent with observations in adult GPP, where IL‐12/23 or IL‐17A blockade can lead to some clinical improvement, but may not fully control inflammation.^9^, ^10^ Similar observations have been reported in psoriasis, where clinically resolved lesions may retain residual immune memory signatures and IL‐17‐producing T cell populations despite treatment‐induced clinical improvement.^11^, ^12^

To further assess the generalisability of our findings, we compared our results with an independent publicly available PBMC scRNAseq dataset from patients with GPP.^13^ Despite differences in patient characteristics and study design, several principal immune alterations, including reduced Treg‐associated programs, enhanced CD8 T cell cytotoxicity programs, and increased inflammatory CD14 monocyte programs were also observed, demonstrating that several principal immune signatures were reproduced across two independent scRNAseq datasets (Figure S2).

Our findings are also complementary to our previous single‐cell analysis of lesional skin from the same paediatric familial GPP patients.^3^ Using the same module scoring approach, Th17‐associated score, cytotoxicity score, and inflammatory myeloid programs demonstrated concordant treatment‐associated trends in lesional skin, with enrichment before treatment and decreased module scores following biologic therapy (Figure S3). While the previous study focused on local cutaneous responses before and after biologic therapy, the present longitudinal PBMC analysis extends these findings by additionally characterising systemic immune alterations in comparison with mutation‐negative sibling controls and longitudinal post‐treatment sampling. Together, these datasets provide complementary insights into local and circulating immune responses in paediatric familial GPP.

Several limitations of this study should be acknowledged, including the small cohort size, the use of different biologic therapies in the two patients which may limit our ability to fully distinguish shared disease‐associated features from treatment‐specific effects, and the lack of orthogonal functional validation. Nevertheless, this study represents, to our knowledge, one of the first longitudinal single‐cell analyses of paediatric familial GPP before and after biologic therapy. Paired pre‐ and post‐treatment sampling and the inclusion of mutation‐negative sibling controls enabled characterisation of systemic immune alterations while partially controlling for shared genetic and environmental factors. In addition, this systemic PBMC‐based analysis complements our previous single‐cell study of lesional skin from the same patients by providing a circulating immune perspective on disease‐associated and treatment‐responsive immune programs. Collectively, these findings suggest that certain immune alterations may persist despite clinical remission following biologic therapy. Such persistent transcriptional programs raise the possibility that immune dysregulation may persist beyond clinical resolution and could contribute to future disease recurrence. Future prospective studies incorporating larger longitudinal cohorts, standardised biologic treatment, and orthogonal protein‐level and functional immune profiling will be important to validate these transcriptomic observations and determine their potential utility as biomarkers of residual immune activation and disease recurrence.

## AUTHOR CONTRIBUTIONS

H.J.K., S.J.J., and J.C. conceived and designed the study, and obtained funding. S.L., S.J.J. and J.C. recruited participants and collected clinical samples. S.J. and C.S.J. performed the experiments and acquired and analysed the data. S.J., S.L., C.S.J., J.G.K., H.J.K., and S.J.J. interpreted the data. H.J.K., S.J.J., and J.C. supervised the study. S.J. drafted the manuscript. All authors critically revised the manuscript and approved the final version.

## CONFLICT OF INTEREST STATEMENT

The authors have no conflict of interest within the scope of the submitted work.

## FUNDING INFORMATION

This research was supported and funded by SNUH Lee Kun‐hee Child Cancer & Rare Disease Project, Republic of Korea (grant number: 22B‐001‐0100); the National Research Foundation of Korea (NRF) grant funded by the Korea government (MSIT) (RS‐2025‐00516052); and a grant of the Korea Health Technology R&D Project through the Korea Health Industry Development Institute (KHIDI), funded by the Ministry of Health & Welfare, Republic of Korea (grant number: RS‐2024‐00403375).

## ETHICS STATEMENT

This study was approved by the Institutional Review Board of Seoul National University Hospital (IRB No. 2210‐035‐1368). The patients in this manuscript have given written informed consent to the publication of their case details.

## Supporting information



Supporting Information

## Data Availability

The data that support the findings of this study are available in GEO under accession number GSE335590.
