# Supplementary material for: Systemic single‐cell analysis of GPP reveals treatment‐responsive and persistent immune signatures
Source: Clin Transl Med. 2026 Aug 3;16(8):e70753. doi: 10.1002/ctm2.70753 (PMC13430424; doi:10.1002/ctm2.70753)
Supplement: Supplementary file 1 — Supporting Information [file CTM2-16-e70753-s001.docx]

**Supporting Information**

**Systemic single-cell analysis of GPP reveals treatment-responsive and persistent immune signatures**

Soyoung Jeong, PhD^1,2^*, Seungbok Lee, MD, PhD^3,4^*, Christine Suh-Yun Joh, BSA^1^, James G Krueger, MD^5^, PhD, Hyun Je Kim, MD, PhD^1,6,7,8,9^†, Seong Jin Jo, MD, PhD^7,10,11^†, Jong-Hee Chae, MD, PhD^3,4^,

^1^Department of Biomedical Sciences, Seoul National University Graduate School, Seoul, Korea

^2^Genomic Medicine Institute, Seoul National University Medical Research Center, Seoul, Korea

^3^Department of Pediatrics, Seoul National University College of Medicine, Seoul National University Children's Hospital, Seoul, Korea

^4^Department of Genomic Medicine, Seoul National University Hospital, Seoul, Korea

^5^Laboratory of Investigative Dermatology, The Rockefeller University, New York, USA

^6^Department of Microbiology and Immunology, Seoul National University College of Medicine, Seoul, Korea

^7^Department of Dermatology, Seoul National University Hospital, Seoul, Korea

^8^Cancer Research Institute, Seoul National University College of Medicine, Seoul, Korea

^9^Interdisciplinary Program in Artificial Intelligence (IPAI), Seoul National University

^10^Department of Dermatology, Seoul National University College of Medicine, Seoul, Korea

^11^Institute of Human-Environmental Interface Biology, Medical Research Center, Seoul National University, Seoul, Korea.

*These authors contributed equally.

^†^Co-corresponding authors

**Supplementary Methods**

**Sample collection and processing**

Peripheral blood samples were obtained from two pediatric patients with familial generalized pustular psoriasis (GPP) carrying *IL36RN* mutations (c.28C>T; p.Arg10Ter and c.115+6T>C; p.Arg10ArgfsTer1) before and after biologic treatment, as well as from two unaffected mutation-negative sibling controls. This study was approved by the Institutional Review Board of Seoul National University Hospital (IRB No. 2210-035-1368).

Peripheral blood mononuclear cells (PBMCs) were isolated using density-gradient centrifugation with Cell Preparation Tubes. Isolated PBMCs were cryopreserved in CellBanker I and stored in liquid nitrogen until use. Prior to single-cell RNA sequencing (scRNAseq), cryopreserved PBMCs were thawed, washed and resuspended in media.

Clinical information, treatment regimens, and sampling time points are summarized in Supplementary Table S1. GPP1 received ustekinumab, and GPP2 received secukinumab according to treatment protocols. Post-treatment (Post Tx) samples were collected at week 28 following treatment initiation in both patients.

**Single-cell RNA sequencing data generation and analysis**

Cells were then loaded onto the Chromium Controller. cDNA generation and gene expression library construction were conducted according to the manufacturer’s protocols (10x Genomics user guide CG000330) using the Chromium Next GEM Single Cell 5’ Kit v2 (PN-1000263) and Library Construction Kit (PN-1000190). The quality of final libraries was checked on Agilent TapeStation 4150 (Agilent Technologies). Libraries were pooled and paired-end sequenced on an Illumina NovaSeq 6000.

Data were aligned to the human reference genome using Cellranger v7.1.0. Further analysis was conducted on R using the Seurat package. SoupX was used to remove ambient RNA. For preprocessing and quality control, cells were filtered using gene counts between 200 and 5,000, and a mitochondrial gene percentage threshold of under ten percent. Doublets were removed using scDblFinder. Harmony integration was conducted to integrate data and remove batch effects. Cells were annotated into broad subsets. Major immune lineages were subsequently subclustered and further annotated using canonical marker genes. Differentially expressed genes (DEGs) were identified using the FindMarkers function. Significant DEGs were defined as genes with adjusted P value < 0.05. UCell was used for gene set module scoring, using curated gene signatures.

To further assess the reproducibility of the principal transcriptomic findings, we also analyzed a publicly available PBMC scRNAseq dataset of patients with GPP and healthy controls (GSE182244)^1^. Publicly available processed expression matrices and accompanying metadata with cell type annotations were obtained, and UCell module scores were calculated using the same gene signatures as those applied in the present study to enable distinct comparison across datasets.

To compare local and systemic immune alterations, we reanalyzed our previously published scRNAseq dataset of lesional skin obtained from the same pediatric familial GPP patients before and after biologic therapy^2^. UCell module scores were calculated using the same curated gene signatures applied to the present PBMC dataset.

**Statistical analysis**

Statistical analysis was conducted using the Wilcoxon rank sum test as implemented in Seurat. Multiple testing correction was performed using the Benjamini-Hochberg method, and adjusted P values < 0.05 were considered significant unless otherwise specified. For gene expression and module score comparisons, individual cells were used as the unit of analysis. To facilitate interpretation at the subject level, sample-level mean values are additionally shown as black dots in the corresponding figures. Given the limited number of available subjects, formal subject-level statistical comparisons were not performed, and the results should be interpreted as descriptive of cell-level patterns.

**References**

1. Haskamp S, Frey B, Becker I, Schulz-Kuhnt A, Atreya I, Berking C, et al. Transcriptomes of MPO-Deficient Patients with Generalized Pustular Psoriasis Reveals Expansion of CD4+ Cytotoxic T Cells and an Involvement of the Complement System. J Invest Dermatol. 2022 Aug;142(8):2149-2158.e10. doi:10.1016/j.jid.2021.12.021 PubMed PMID: 34973310.

2. Jeong S, Joh CSY, Lee S, Krueger JG, Chae JH, Kim HJ, et al. Single-cell transcriptomic analysis of GPP patients treated with IL-12/23 or IL-17A blockade. J Eur Acad Dermatol Venereol JEADV. 2025 Oct;39(10):e925–8. doi:10.1111/jdv.20659 PubMed PMID: 40130948.


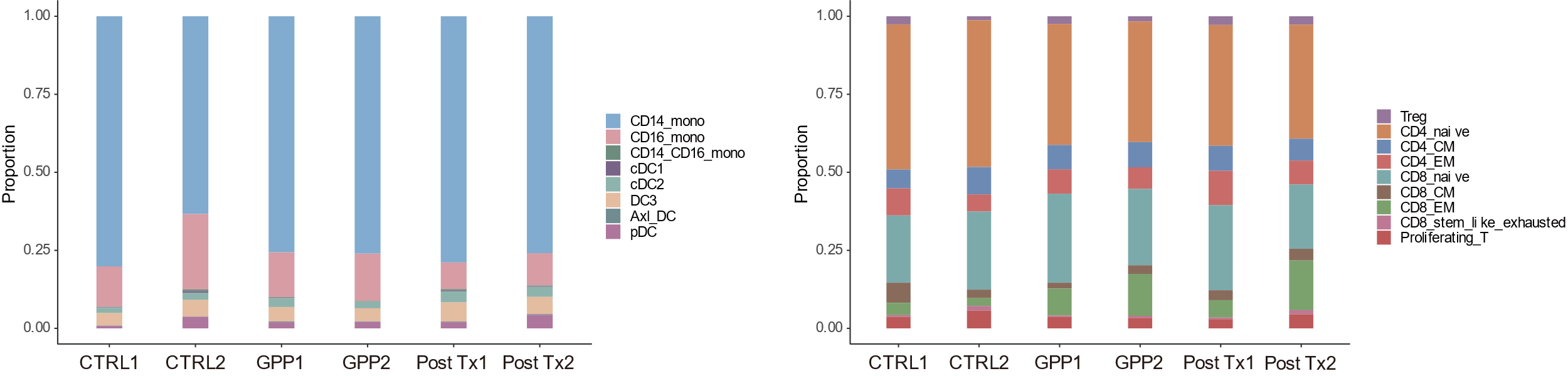


Supplementary Figure S1. Cell type proportions by sample (in relation to Figure 1e and 2a). Stacked bar plots showing cell type proportions. DC and monocyte subsets are shown as a percentage of total DC and monocytes for each sample (left). Proportions of T cell subsets are shown as a percentage of total T cells (right).


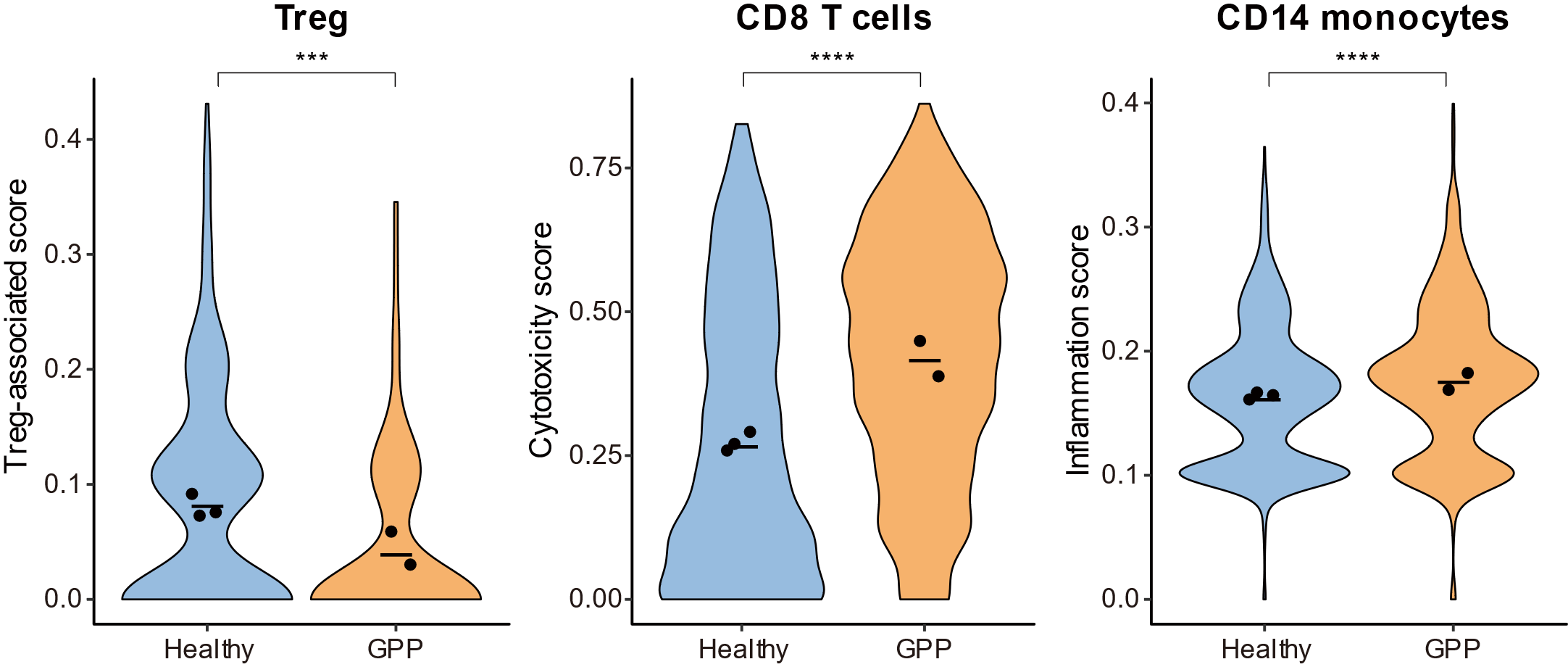


Supplementary Figure S2. Comparison of gene signatures in an independent cohort. Violin plots summarizing UCell scores in healthy control (n=3) and GPP (n=2) PBMC samples (GSE182244). Scores were calculated using UCell based on the gene lists: Treg-associated score (*FOXP3*, *IL2RA*, *CTLA4*, *IKZF2*, *TIGIT*, *TNFRSF18*, *TGFB1*) in Tregs, cytotoxicity score (*PRF1*, *GZMB*, *GNLY*, *GZMH*, *TBX21*, *FCGR3A*, *CCL4*, *CCL5*) in CD8 T cells, inflammation score (*IL1B*, *CXCL8*, *S100A8*, *SERPINB1*, *TNF*, *MARCO*, *CCL2*, *CD86*, *CD40*, *IL1RN*) in CD14 monocytes.


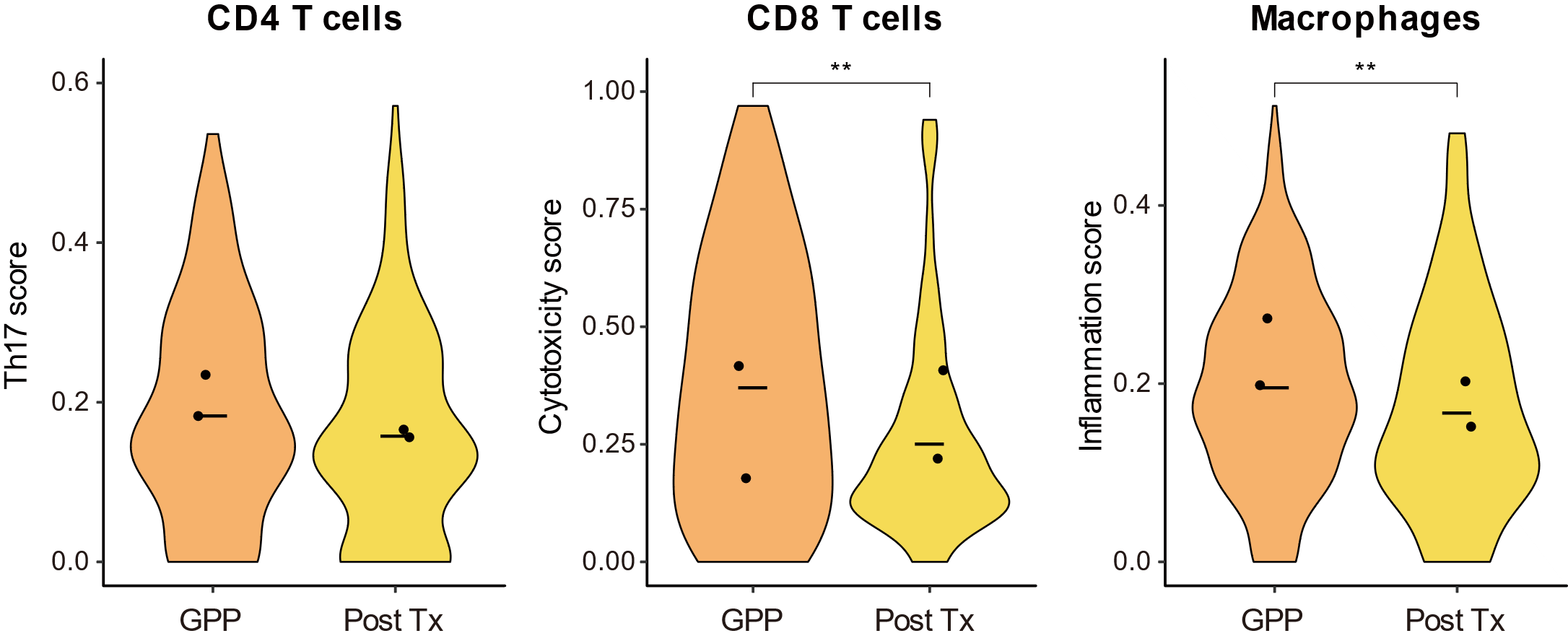


Supplementary Figure S3. Comparison of systemic signatures with tissue signatures. Violin plots summarizing UCell scores in pre- and post-treatment GPP tissues. Scores were calculated using UCell based on the gene lists: Th17 score (*RORA*, *RORC*, *STAT3*, *CCR4*, *CCR6*, *IL17A*) in CD4 T cells, cytotoxicity score (*PRF1*, *GZMB*, *GNLY*, *GZMH*, *TBX21*, *FCGR3A*, *CCL4*, *CCL5*) in CD8 T cells, inflammation score (*IL1B*, *CXCL8*, *S100A8*, *SERPINB1*, *TNF*, *MARCO*, *CCL2*, *CD86*, *CD40*, *IL1RN*) in macrophages. Similar to systemic signatures, all three scores that were high in GPP decreased after treatment.

**Supplementary Table S1. Clinical characteristics of study participants**

| Subject | Group | Sex | Age (yr) | Mutation | Treatment | Treatment regimen | Sampling time | Baseline GPPGA | Post Tx GPPGA |
| --- | --- | --- | --- | --- | --- | --- | --- | --- | --- |
| GPP1 | GPP | M | 11 | c.28C>T; p.Arg10Ter and C.115+6T>C; p.Arg10Argfs Ter1 | Ustekinumab | 45 mg at weeks 0, 4, 16 | Baseline, week 28 | 12.6 | 0 |
| GPP2 | GPP | F | 4 | c.28C>T; p.Arg10 Ter and C.115+6T>C; p.Arg10Argfs Ter1 | Secukinumab | 75 mg at weeks 0, 1, 2, 3, 4, then every 4 weeks | Baseline, week 28 | 13.4 | 0 |
| CTRL1 | Control | M | 8 | Mutation-negative | N/A | N/A | Baseline | N/A | N/A |
| CTRL2 | Control | F | 2 | Mutation-negative | N/A | N/A | Baseline | N/A | N/A |

CTRL, control; F, female; GPP, generalized pustular psoriasis; GPPGA, generalized pustular psoriasis physician global assessment; M, male; N/A, not applicable; Post Tx, post-treatment.
